# Treatment of Endovascular Coil and Stent Migration Using the Merci Retriever: Report of Three Cases

**DOI:** 10.1155/2012/242101

**Published:** 2012-05-17

**Authors:** David K. Kung, Taylor J. Abel, Karthik H. Madhavan, Richard T. Dalyai, Brian J. Dlouhy, Wei Liu, Pascal M. Jabbour, David M. Hasan

**Affiliations:** ^1^Department of Neurosurgery, University of Iowa Hospitals and Clinics, Iowa City, IA 52242, USA; ^2^Department of Neurological Surgery, Thomas Jefferson University Hospital, Philadelphia, PA 19107, USA; ^3^Division of Interventional Neuroradiology, Department of Radiology, University of Iowa Hospitals and Clinics, Iowa City, IA 52242, USA

## Abstract

*Background.* Coil and stent migration is a potentially catastrophic complication in endovascular neurosurgery, which may lead to cerebral thromboembolism. Techniques for removing migrated coil and stent are not well established. *Methods and Results.* We present three cases in which coil or stent migration occurred during endovascular embolization of a cerebral aneurysm. The Merci Retrievers were used successfully in all cases to remove the displaced foreign bodies. Technical details are described. *Conclusion.* The Merci Retriever device can be utilized successfully for removal of migrated coils and stents in endovascular neurosurgery.

## 1. Introduction

Occlusion of cerebral aneurysms by detachable coils through the endovascular approach has gained significant popularity over the last two decades and is now a common approach for securing cerebral aneurysms [1–3]. Migration of coils or stents from their targets represents one of the most challenging complications of endovascular neurosurgery and contributes significantly to thromboembolic events following endovascular embolization [4]. No “gold standard” method has been identified for retrieval or repositioning of the migrated foreign bodies, but several different techniques and devices are described in the literature [5–9]. The Merci Retriever device is FDA approved for percutaneous mechanical thrombectomy in patients who have suffered ischemic stroke [10, 11]. Case reports have demonstrated its use for removal of foreign bodies during endovascular embolization of cerebral aneurysms [12–14]. Here, we present use of the Merci Retriever for correction of coil prolapse and stent misplacement in three patients undergoing endovascular coil embolization of cerebral aneurysms. Additionally, we review the literature describing this use of the Merci Retrieval device.

## 2. Case Report

### 2.1. Case 1

**Figure 1:**
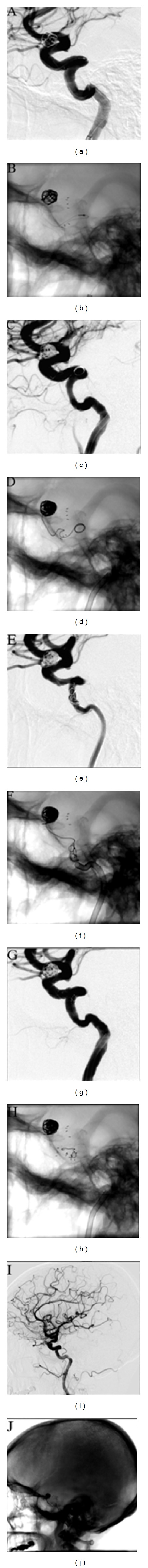
(a) and (b) Left carotid artery injection, lateral view; and plain X-ray. The stent was placed across the aneurysm neck and initial coil embolization performed. (c)–(f) During further coil embolization, the coil detached from the delivery catheter without activating the release mechanism. Manipulation and attempts to retrieve the misplaced coil with the Alligator and Microsnare device resulted in further coil herniation. (g) and (h) Left carotid artery injection, lateral view, and plain X-ray demonstrate that the misplaced coil was removed by the Merci device; a small fragment is left in the cavernous ICA and jailed to the wall with a second stent. (i) and (j) Angiogram and plain X-ray obtained after endovascular treatment show normal filling of the ICA and aneurysm occlusion.

 A 53-year-old woman presented with subjective sensation of retroorbital pulsation. Digital subtraction angiogram demonstrated a 10 mm left supraclinoid ophthalmic artery aneurysm with a 5.5 mm neck. She underwent elective stent-assisted coiling of this aneurysm. The procedure was performed under general anesthesia. Clopidogrel 600 mg and aspirin 325 mg was given through a nasogastric tube at the beginning of the case. Femoral artery access was established with a 7 F 11 cm sheath. Heparin was given intravenously to maintain activated coagulation time between 250 and 300 seconds throughout the case. A Neuroform-3 (4 × 20 mm) stent (Boston Scientific, Natick, MA, USA) was deployed across the aneurysm neck. The aneurysm was then embolized using two HydroFrame coils (MicroVention, Aliso Viejo, CA, USA) through an Excelsior SL 10 microcatheter (Boston Scientific, Natick, MA, USA) positioned within the aneurysm dome (Figures 1(a) and 1(b)). A Hydrosoft 10 helical 4 × 6 mm coil (MicroVention, Aliso Viejo, CA, USA) was then chosen to continue the embolization. During delivery the coil detached from the delivery system without activation of the release mechanism. A long portion of the coil was left in the cavernous internal carotid artery (ICA) proximal to the aneurysm (Figures 1(c) and 1(d)). Retrieval of the displaced coil was first attempted using the Alligator 4 mm Retrieval Device (ev3 Endovascular, Inc, Plymouth, MN, USA), but this was unsuccessful. An Amplatz 4 mm GooseNeck Microsnare (ev3 Endovascular, Inc, Plymouth, MN, USA) was then used for retrieval, but this resulted in further coils prolapse (Figures 1(e) and 1(f)). At this point the ICA developed significant vasospasm. Nicardipine was infused intra-arterially with radiographic improvement. Abciximab (Reopro, Centocor, Malvern, PA, USA) 0.125 mcg/kg/min was also started to prevent thromboembolic complication. A Merci V 2.5 Soft retriever (Concentric Medical Inc, Mountain View, CA, USA) was advanced to the displaced coil and engaged the coil loops. Multiple fragments of the coil were retrieved. A remaining coil fragment was jailed to the vessel wall by placement of another Neuroform 3 stent (Figures 1(g) and 1(h)). A 6 French Envoy guide catheter was used in the ICA, and this was removed simultaneously with the Merci device (Codman & Shurtleff Inc, Raynham, MA, USA). Post-operative angiogram shows near complete occlusion of the aneurysm. Flow in the ICA and its branches were normal (Figures 1(i) and 1(j)). Abciximab was stopped 6 hours after the procedure. The patient had an uneventful postoperative course and was discharged to home neurologically intact. On three-month followup the patient remains neurologically normal (modified Rankin score = 0); and she has 20/20 vision bilaterally. She reported significant improvement in her subjective retroorbital pulsation. Magnetic resonance angiogram 1 year after procedure shows no evidence of aneurysm recurrence or coil compaction.

### 2.2. Case 2

**Figure 2:**
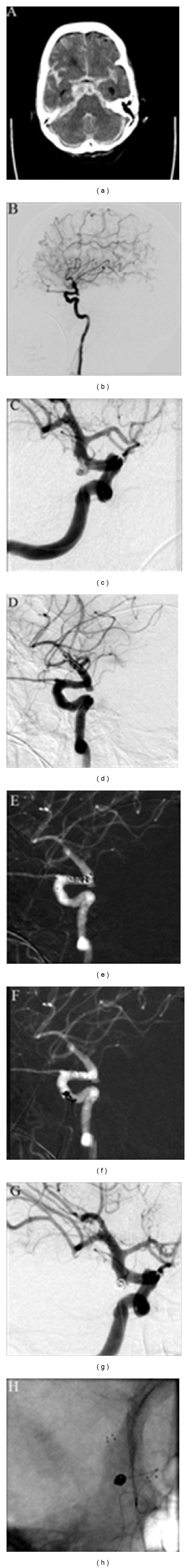
(a) Noncontrast head CT demonstrates diffuse SAH. (b) A lateral view of the right ICA arteriogram reveals a wide-necked PCOM aneurysm. (c) Right ICA injection angiogram at the working angle demonstrates partial coil prolapse following initial stent-assisted coiling. (d) Attempts to place an overlapping stent to hold the prolapsed coil in place resulted in anterior migration of the stent and complete coil herniation. (e) and (f) Lateral roadmap angiograms demonstrate sequential retrieval of the stent and coil mass. (g) and (h) After complete removal of the migrated stent and coils, a repeated attempt at stent-assisted coiling of the ruptured aneurysm is successful. A working view of left ICA angiogram and plain X-ray demonstrate final placement of the stent and coil.

 A 75-year-old woman with atrial fibrillation presented with Hunt Hess grade four subarachnoid hemorrhage (SAH). Digital subtraction angiography revealed a 2.5 mm right posterior communicating artery (PCOM) aneurysm with irregular dome (Figures 2(a) and 2(b)). She underwent emergent ventriculostomy and stent-assisted coiling of the ruptured aneurysm. Femoral artery access was established with a 7 F 11 cm sheath. Heparin was not given. A Prowler Select Plus microcatheter (Cordis Endovascular, Miami Lakes, Florida, USA) led by a Synchro 2 soft microwire (Boston Scientific, Natick, MA, USA) was advanced into the distal ICA. An Enterprise 4.5x22 mm stent (Cordis, Bridgewater Township, NJ, USA) was deployed across the neck of the aneurysm. Migration of the stent was noted during deployment, but it still covered the neck of the aneurysm. Tirofiban 0.1 microgram/kg/min was started intravenously. Two HydroCoil 10 coils (2 mm × 4 cm) (MicroVention, Aliso Viejo, CA, USA) were initially deployed into the aneurysm through a SL-10 microcatheter (Boston Scientific). During coiling embolization several loops of the coil bulged into the parent artery (Figures 2(c) and 2(d)). A decision was made to deploy an overlapping stent to hold the coil mass in the aneurysm. A Renegade Hi-Flo microcatheter (Boston Scientific, Natick, MA, USA) led by a Synchro 2 soft microwire was advanced into the vasculature. Significant migration of the previously placed stent was noted when the microcatheter and microwire passed through the Enterprise stent and the coil mass herniated out of the aneurysm (Figures 2(e) and 2(f)). Attempts to engage the stent and coils using the Synchro 2 soft microwire were without success. A Merci V 3.0 firm Retriever was advanced into the ICA led by an 18 L Merci microcatheter (Concentric Medical Inc, Mountain View, CA, USA). We were able to engage the coils and stent and pulled them into the cavernous segment. A Merci V 2.5 soft Retriever was then advanced to the ICA. The Retriever engaged the coils and the stent again but attempts to pull them into the 6 F Envoy MPD guiding catheter (Codman & Shurtleff Inc, Raynham, MA, USA) were unsuccessful. At this point the coil/stent mass dislodged from the Merci device. The Merci Retriever, the 18 L Merci microcatheter, and the 6 F guiding catheter were then removed. A 7 F Envoy catheter led by a 0.038 glidewire was then used to select right carotid artery, and the tip of guiding catheter was placed at the distal cervical ICA near petrous bone. The 18 L Merci microcatheter led by a Synchro 2 soft microwire was again advanced into the distal ICA. The microwire was then removed, and a Merci V 2.0 Firm Retriever was advanced. The coils and the stent were pulled to the tip of the guiding catheter. The guiding catheter, the microcatheter, and the Retriever along with the coils and the stent were successfully removed out of the sheath. Examination of the objects on the field showed that the stent and the coils were intact without missing pieces. A postretrieval angiogram shows that the aneurysm was stable and the right ICA was patent without evidence of arterial dissection, pseudoaneurysm, or occlusion.

 A subsequent attempt at stent-assisted coiling of the aneurysm was successful using a Neuroform 4.0 mm × 20 mm stent (Boston Scientific, Natick, MA, USA) and HydroCoils. A control angiogram after final coil deployment shows obliteration of the aneurysm (Figures 2(g) and 2(h)). The stent was patent. An intracranial run showed that the distal ICA and its branches were normal in caliber without vasospasm or thromboembolism. Clopidogrel 600 mg and aspirin 325 mg was given at the end of the procedure. A repeat angiogram 3 days later showed moderate vasospasm and a secured PCOM aneurysm. Unfortunately the patient's clinical exam did not improve significantly, and the family decided on palliative care. She expired one month after the initial hemorrhage.

### 2.3. Case 3

**Figure 3:**
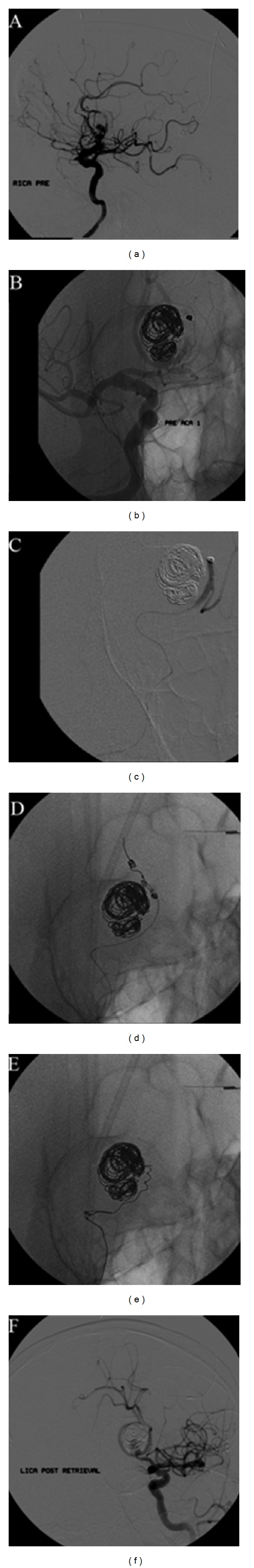
(a) Lateral view of left carotid injection demonstrates recanalization of the previously coiled ACOM aneurysm. (b) Nonsubtracted angiogram at the working view shows the dislodged coil in the left A2 segment. (c) Superselective injection demonstrates that no distal flow passes the migrated coil. (d) Plain X-ray demonstrating the Merci device passing the coil obstruction. (e) The Merci device ensnares the migrated coil. (f) Postretrieval angiogram shows reconstitution of distal left A2 flow.

A 68-year-old man presented in 2010 with recanalization of a previously treated anterior communicating artery (ACOM) aneurysm (Figure 3(a)). He had a history of Grade I SAH in 2004 from a ruptured 15 mm ACOM aneurysm. He was treated initially with endovascular coiling in 2004, and a repeated coil embolization was performed in 2008. A decision was made to treat this recurrent aneurysm with further coiling. Femoral artery access with a 7 F sheath was obtained. A 6 F Envoy guide catheter was introduced into the left ICA over a guidewire. Superselectively, the aneurysm was catheterized with a SL-10 microcatheter and a Synchro 10 microwire. A 3 × 6 mm Orbit Galaxy coil (Codman & Shurtleff Inc, Raynham, MA, USA) was deployed in the aneurysm, but not detached since the coil was oversized and herniated in the parent vessel. Subsequently, a 2 × 4 mm Galaxy Xtrasoft coil (Codman & Shurtleff Inc, Raynham, MA, USA) was deployed in the aneurysm. As soon as it was detached, the flow carried the coil from the aneurysm to the left A2 segment of the anterior cerebral artery (Figure 3(b)). A repeated injection demonstrates thrombosis of the left A2 without flow distal to the migrated coil (Figure 3(c)). At this point the SSEP signals were lost from the right leg. The decision was made to retrieve the coil with a Merci retriever, but the angle of the left A1/A2 junction was too acute to allow delivery of the Merci device from the left. An exchange maneuver was performed to replace the femoral access with an 8F sheath. An 8F Merci balloon guide catheter (Concentric Medical Inc, Mountain View, CA, USA) was also exchanged to replace the 6 F Envoy catheter and placed in the right ICA with the aim to diminish flow by inflating the balloon at the tip of the Merci guide while we are retrieving the coil. The left A2 was then catheterized through the right A1 with an 18 L Merci microcatheter. The microcatheter was advanced proximal to the migrated coil first, and 8 mg of tissue plasminogen activator was infused. The catheter was then taken distal to the coil, and a V2.0 soft Merci retriever was deployed and successfully ensnared the migrated coil (Figures 3(d) and 3(e)). With the proximal balloon inflated, the coil was brought down to the tip of the guide catheter. At this point, we were unable to bring the coil inside the guide catheter, so the Merci retriever was removed, and an Alligator 2 mm Retrieval Device (ev3 Endovascular, Inc, Plymouth, MN, USA) was utilized to grab the coil and bring it inside the guide catheter. Postprocedure angiography demonstrates patency of the Left A2 (Figure 3(f)). At this point the SSEP signals returned to normal. Postoperatively the patient remains neurologically intact and was scheduled for a repeated coiling in the future.

## 3. Discussion

 With the widespread use of endovascular treatment for aneurysms comes the new consideration of retrieving dislodged foreign bodies intravascularly. Even in Guglielmi's original clinical series, coil migration into the parent artery was a recognized possibility, but it was considered only prior to coil deployment when it could be readily corrected [2]. The risk of coil migration is influenced by anatomical, flow, and technical factors. Coil migration risk is thought to increase as the width of the aneurysm neck increases. The risk of coil prolapse can be mitigated when treating wide-neck aneurysms by the use of a stent- or balloon-assisted technique [15]. Tortuous vessels and high-flow conditions are also thought to increase the risk coil migration [6]. On the technical side, premature coil deployment and the use of undersized coils are the significant factors for coil migration. When prolapsed from the aneurysm, the coil acts as a conduit for aggregation of blood components ultimately leading to thrombosis and possibly distal thromboembolism [3, 4].

Coil migration is not an uncommon occurrence and increase the risk of cerebral thromboembolism. A retrospective study of 1811 endovascularly treated aneurysms demonstrated coil migration in 2.5% [16]. Early studies suggested that prolapsed coils increases the risk for ischemic events following endovascular aneurysm treatment [17, 18], a suspicion that was later confirmed by Derdeyn et al. [4]. In their study of 178 consecutive patients, Derdeyn et al. found that aneurysm size and coil protrusion were the only two variables with significant association to postprocedure ischemic events [4]. Of these patients, there were 21 occurrences of coil prolapse, which includes four of the nine patients with postprocedure ischemic events.

Because of the increased risk for thromboembolism, once detected, coil migration should be managed immediately. Systemic heparinization during coil placement confers protection against cerebral thromboembolism in the immediate period. Definitive treatment, however, is removal of the coil from the parent artery. A number of techniques have been derived, but none have gained acceptance as a gold standard [6]. Prior to deployment, the coil or stent can simply be retracted by the delivery device and repositioned [2]. Microsnares have been employed in some instances of coil migration, including coil migration in the MCA and distal ACA [5, 19, 20]. Microsnare retrieval can be accomplished in vessels with a >3 mm diameter; however if the vessel diameter makes the loop opening difficult, then the use of the microsnare carries a risk of dissection or perforation [6]. The Alligator Retrieval Device has recently been introduced and used for both coil [6] retrieval and retrieval of misplaced stents [21]. Stent deployment has been utilized as a means of trapping migrated coils into the aneurysm sac or against the vessel wall with promising results [15, 22]. Additionally, partial stent deployment has been used to removed herniated coils [23]. Open surgery may also be an option in cases where endovascular retrieval is not possible, but this is obviously more invasive and not necessarily more effective [24–29].

 A review of the literature reveals several reports of the Merci Retriever device utilized for the management of coil migration [13, 14, 30, 31], which are summarized in Table 1 along with the cases presented here.

 Vora et al. [14] reported the case of a 37-year-old man who suffered a subarachnoid hemorrhage from vertebral confluence aneurysm. During treatment of the aneurysm by stent-assisted coil embolization, a misplaced coil became entangled with the stent during attempted repositioning. The Merci device was utilized successfully for removal of the stent-coil complex, and the aneurysm was subsequently embolized. O'Hare and colleagues [13] reported migration of the coil from a PCOM aneurysm to the MCA, which once free in the MCA was successfully retrieved using an old-generation X6 Merci Retriever.

 O'Hare and colleagues reported unsuccessful use of the Merci Retriever to retract a coil prolapsed through a Neuroform 3 stent [31]. In this scenario, the Merci Retriever was successful in grasping the misplaced coil; however the coil became caught on the stent at the time of retraction. The authors were not successful in removing this coil. It is likely that there have been other cases of unsuccessful use of the Merci Retriever that have not been reported, and more research is necessary to determine the role the Merci Retriever will play in the treatment of coil migration complications.

## 4. Conclusions

 Misplacement of intracranial coils or stents remains a potential hazard in endovascular neurosurgery. The Merci device provides a secure technique for retrieval of herniated coils. Our experience demonstrates that the Merci device can be used successfully to rescue patients with this potentially devastating complication.

## Figures and Tables

**Table 1:** 

Author/yr	Pt Age/sex	Clinical presentation	Aneurysm location	Retrieval device—success?	Outcome
Vora et al., 2008 [14]	37/m	LOC, SAH, HH = IV	Vertebral confluence	Merci L5—success	Brain death.
O'Hare et al., 2009 [13, 31]	54/m	HA, SAH, HH = I	R PCOM	Merci X6—success	No neurologic deficit.
O'Hare et al., 2009 [13, 31]	43/f	Incidental diagnosis	L periophthalmic	Merci—unsuccessful retrieval	R hand extensor weakness.
Case #1	53/f	Incidental diagnosis	L supraclinoid	Merci V2.5—success	No neurologic deficit.
Case #2	75/f	SAH, HH = IV	R PCOM	Merci V3, V2.5, and V2.0—success	Death at 1 month from respiratory failure.
Case #3	68/m	Recurrent aneurysm	ACOM	Merci V2.0—success	No neurologic deficit.

m: male, f: female, R: right, L: left, LOC: loss of consciousness, SAH: subarachnoid hemorrhage, HA: headache, HH: Hunt & Hess, PCOM: posterior communicating artery, and ACOM: anterior communicating arter.
